# Cell of origin and mutation pattern define three clinically distinct classes of sebaceous carcinoma

**DOI:** 10.1038/s41467-018-04008-y

**Published:** 2018-05-14

**Authors:** Jeffrey P. North, Justin Golovato, Charles J. Vaske, J. Zachary Sanborn, Andrew Nguyen, Wei Wu, Benjamin Goode, Meredith Stevers, Kevin McMullen, Bethany E. Perez White, Eric A. Collisson, Michele Bloomer, David A. Solomon, Stephen C. Benz, Raymond J. Cho

**Affiliations:** 1 Department of Dermatology, University of California, San Francisco, CA 94115 USA; 2 Department of Pathology, University of California, San Francisco, CA 94143 USA; 3 NantOmics, LLC, Culver City, CA 90232 USA; 4 Skin Tissue Engineering Core and Department of Dermatology, Feinberg School of Medicine, Northwestern University, Chicago, IL 60611 USA; 5 Department of Hematology and Oncology, University of California, San Francisco, CA 94158 USA; 6 Department of Ophthalmology, University of California, San Francisco, CA 94143 USA

## Abstract

Sebaceous carcinomas (SeC) are cutaneous malignancies that, in rare cases, metastasize and prove fatal. Here we report whole-exome sequencing on 32 SeC, revealing distinct mutational classes that explain both cancer ontogeny and clinical course. A UV-damage signature predominates in 10/32 samples, while nine show microsatellite instability (MSI) profiles. UV-damage SeC exhibited poorly differentiated, infiltrative histopathology compared to MSI signature SeC (*p* = 0.003), features previously associated with dissemination. Moreover, UV-damage SeC transcriptomes and anatomic distribution closely resemble those of cutaneous squamous cell carcinomas (SCC), implicating sun-exposed keratinocytes as a cell of origin. Like SCC, this UV-damage subclass harbors a high somatic mutation burden with >50 mutations per Mb, predicting immunotherapeutic response. In contrast, ocular SeC acquires far fewer mutations without a dominant signature, but show frequent truncations in the ZNF750 epidermal differentiation regulator. Our data exemplify how different mutational processes convergently drive histopathologically related but clinically distinct cancers.

## Abstract (web summary)

Sebaceous carcinomas (SeC) are cutaneous malignancies that sometimes metastasize and cause death. Here the authors perform whole-exome sequencing on 32 SeC and report distinct mutational classes that may explain cancer ontogeny and clinical outcome.

## Introduction

Sebaceous carcinoma (SeC) accounts for 0.7% of skin cancers and carries a cancer-specific mortality rate of 3–6.7%^1, 2^. SeC are believed to arise from sebaceous glands, ostensibly explaining their occurrence in sebocyte-dense sites such as the eyelids and head and neck regions^3^. Poorly differentiated ocular SeC has been reported to metastasize more frequently, often with fatal outcomes^4^. In all locations, SeC is considered a potential cutaneous marker for Muir–Torre disease, a cancer syndrome associated with germline mutations in mismatch-repair pathway components *MLH1*, *MSH2*, *MSH6*, and *PMS2*^5–7^. Muir–Torre is a variant of hereditary non-polyposis colon cancer, also known as Lynch syndrome, and causes patients to develop endometrial, ovarian, gastric, biliary, and genitourinary tract cancers, as well as benign and malignant sebaceous neoplasms and keratoacanthomas. Tumors defective in this pathway show errors in replicating microsatellite repeat sequences, generating a mutagenesis pattern known as microsatellite instability (MSI).

Recently, Tetzlaff et al.^8^ applied targeted sequencing in ocular SeC, reporting that 52% (14 of 27) harbored somatic mutations in PI3K signaling components. *TP53* and *RB1* mutations were detected in ocular SeC, while four extra-ocular SeC showed additional mutations affecting DNA repair and chromatin remodeling pathways. Three extra-ocular SeC demonstrated a high level of MSI with somatic mutations in the mismatch-repair genes *MLH1* and *MSH2*, suggesting that mutations outside the germline may drive mutation and tumorigenesis in these tumors. However, because of the targeted nature of the sequencing in this study, the broader patterns of mutation in SeC remain unknown.

In this study, we report that SeC fall into three distinct subtypes based on mutational genetics, and are heavily influenced by anatomic site of origin. Two mutually exclusive classes are dominated by either mismatch-repair-derived insertions and deletions or ultraviolet (UV) signature single-nucleotide mutations. In contrast, ocular SeC harbor fewer mutations but acquire recurrent truncating mutations in the *ZNF750* transcription factor.

## Results

### Whole-exome sequencing of 32 SeC

**Fig. 1:**
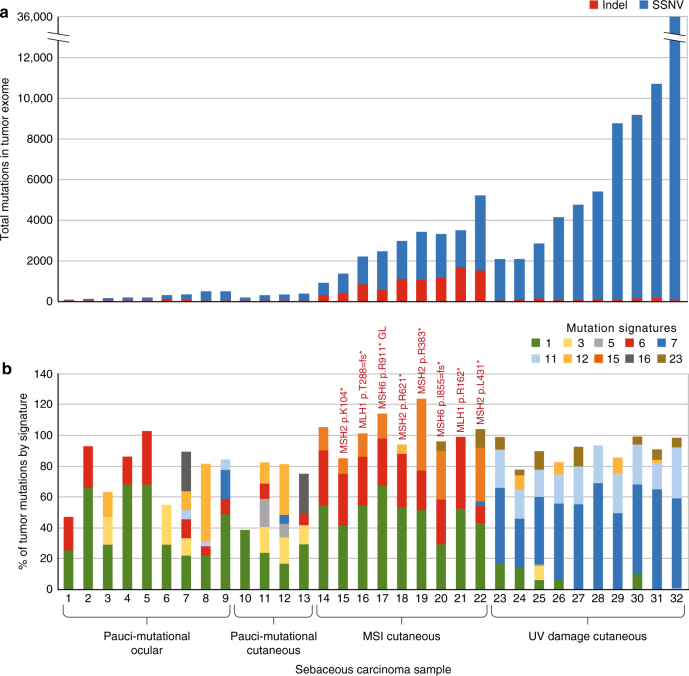
Sebaceous carcinomas are distinguished by somatic mutation burden and type into pauci-mutational, MSI, and UV-damage categories. **a** Total numbers of SSNVs (blue) and indels (red) are shown for each of 32 SeC in this study. Pauci-mutational SeC (samples 1–13) show a mutation rate ranging from 1.2 to 5.2 per Mb, while MSI and UV tumors reach rates of 82.6 and 586 per Mb, respectively. Nine of the 13 pauci-mutational tumors (samples 1–9) represent all 9 ocular SeC sequenced in this report. **b** The percentage of mutations demonstrating each of the Stratton mutational signatures are shown, for signatures present in at least 10% of mutations of at least one sample. High frequency of DNA damage mutations (signatures 6 and 15 in red and orange, respectively) vs. UV-damage signatures (7 and 11 in darker blue and lighter blue, respectively) distinguish SeC. The color key further details other mutational signatures in these samples. While mutations associated with normal aging (signature 1) are sparse in the UV subclass, they represent the single largest class detected in the pauci-mutational cancers. Somatic mismatch repair mutations in MSI tumors are labelled

In order to study the genetic alterations that characterize SeC, we performed whole-exome sequencing and selected transcriptome analysis on a cohort of 32 SeC. To identify possible associations between mutation profile and anatomic site, 23 SeC were sequenced from the head and neck (nine of which were ocular) and 9 were analyzed from the trunk. Supplementary Data 1 details all samples used for this study. Sequencing was performed after targeted library capture of 62.52 Mb of coding nucleotides. The average reads for tumor and normal samples were 2,230,611,589 and 117,499,836, respectively, generating an average coverage of ×84 and ×46 across 99% of captured sequence. The number of somatic single-nucleotide variants (SSNVs) identified in SeC exomes ranged unexpectedly widely, from 73 to 36,659 (Fig. 1a), reflecting mutation prevalences from 1.2 to 536 mutations per Mb (Supplementary Data 2). We found that SeC showed low incidence of CNVs; only 12/32 harbored more than five events. The most common aberration, single-copy loss of chromosome 17p, where *TP53* is located, occurred in nine tumors. No biallelic deletions or focal high-level amplifications were identified. Supplementary Data 3 lists all copy-number aberrations >2.5 megabases in size.

### Mutational classification of sebaceous carcinoma

Thirteen SeC, including all nine ocular SeC, demonstrated a low mutation prevalence between 1.2 and 5.2 per Mb, distinguishing them sharply from the high mutation burden in other tumors (samples 1–13, Fig. 1a). Of the remaining 19 tumors, 9 (28% overall) exhibited a somatic mutation pattern composed of at least 30% indels, while the other 10 highly mutated cancers were predominated by SSNVs (samples 14–22 vs. 23–32, Supplementary Data 2). None of the patients in our study carried known germline mismatch-repair defects, but it was possible that their SeC marked an initial manifestation of Muir–Torre syndrome. We thus analyzed uninvolved normal skin in these patients to assess for possible germline mutations in *MLH1*, *MSH2*, *MSH6*, and *PMS2*, DNA repair genes whose inactivation is causative of Muir–Torre syndrome. A germline nonsense mutation was found in only one case, SeC sample 17 (*MSH6* p.R911*), which harbored high numbers of indels. Truncating mutations or likely damaging mutations in *POLE* or *APOBEC*, other potential sources of high mutation burden, were not detected.

These observations led us to suspect that three distinct mutational mechanisms had given rise to the tumors of our cohort. We therefore examined our mutations using established algorithms capable of distinguishing known mutagenic processes^9^. This analysis structure assesses a somatically altered base and surrounding sequence context to deduce a likely cause of each mutation. These deduced mutational origins are themselves classified into a series of discrete categories or “signatures.” This analysis revealed that the tumors in our study fall into three distinct classes (Fig. 1b). The 9/32 tumors harboring high proportions of indels all showed substantial MSI signatures, with >30% of mutations matching mutational signatures 6 or 15 as defined by the Signatures of Mutational Processes in Human Cancer database (http://cancer.sanger.ac.uk/cosmic/signatures). These mutation patterns are intimately associated with primary defects in DNA mismatch-repair genes. To support these findings, we additionally analyzed an average of 1492 microsatellite sequences (homopolymeric repeats) in the tumor and an average of 1352 in the normal, and found that six of these tumors showed instability in >15% of examined loci^10^. Notably, we found seven of nine of these tumors had acquired truncating somatic mutations in *MLH1*, *MSH2*, or *MSH6*, excluding the tumor with germline *MSH6* mutation (Fig. 1b). Isolated mutation of the mismatch-repair genes *MSH3* or *PMS1* were not observed in our series, consistent with past reports that defects in these genes do not cause Muir–Torre syndrome^11^.

Ten different tumors were defined by mutations harboring a UV-damage signature (signatures 7 and 11) with high numbers of CC > TT dinucleotide mutations induced by exposure to ultraviolet radiation. These SeC developed on sun-exposed areas and showed scarce evidence of mismatch-repair errors (Fig. 1b), averaging a mean of <3% of signatures 6 and 15 combined. In contrast to recent work that attributed high mutation prevalence mainly to mismatch-repair-defective tumors^8^, the UV-damage class tumors in our series showed the most SSNVs, ranging from 15.4 to 586 mutations per Mb. There was limited overlap between the mutation signatures of tumors derived from MSI and UV radiation, suggesting that most SeC arise from a single primary mutational mechanism. Only sample 22, located on the forehead, harbored both MSI and UV signatures, with 46% of mutations classified as consistent with signature 6 or 15, and 3% consistent with signature 7.

**Fig. 2:**
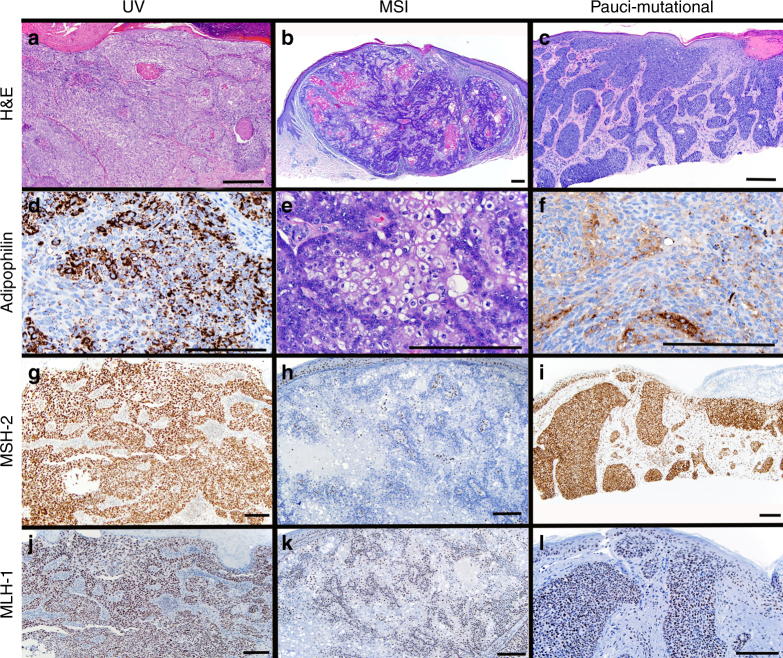
Adipophilin and mismatch repair protein expression distinguish subtypes of sebaceous carcinoma. UV tumors tend to be more poorly differentiated (**a**), but adipophilin staining confirms sebocytic differentiation (**d**). MSI tumors tended to be well-differentiated with clear sebocytic differentiation easily identifiable on routine hematoxylin/eosin staining (**b,e**). This MSI tumor had loss of MSH-2 expression detectable by immunostaining (**h,k**). Pauci-mutational SeC can manifest both moderately differentiatiated (**c,f**) and well differentiated phenotypes (not shown). Both MSH-2 and MLH-1 expression is preserved in UV (**g,j**) and pauci-mutational SeC (**i,l**). Scale bar 0.1 mm.

Interestingly, the remaining 13 of our SeC, including all nine ocular cancers, were pauci-mutational (the group described above with mutation prevalences between 1.2 and 5.5 per Mb), a phenomenon occurring much less frequently in other cancers of sun-exposed skin^12^. This range is on  average 30-fold fewer than in the MSI and UV classes. Tumor purity, estimated based on mutant allele frequency, exceeded 70% in these cancers, confirming an authentically low mutation prevalence. While almost all tumors (88%) showed some amount of signature 1 mutation, eight of the pauci-mutational tumors were found to have SSNVs primarily consistent with this signature, which is typified by spontaneous deamination of 5-methylcytosine and thought to be related to natural aging. Staining of six UV class and six pauci-mutational SeC revealed MLH1 and MSH2 in all 12 tumors, confirming these subgroups maintain expression of mismatch-repair proteins (Fig. 2).

### UV-damage SeC show more aggressive histopathologic features

**Fig. 3:**
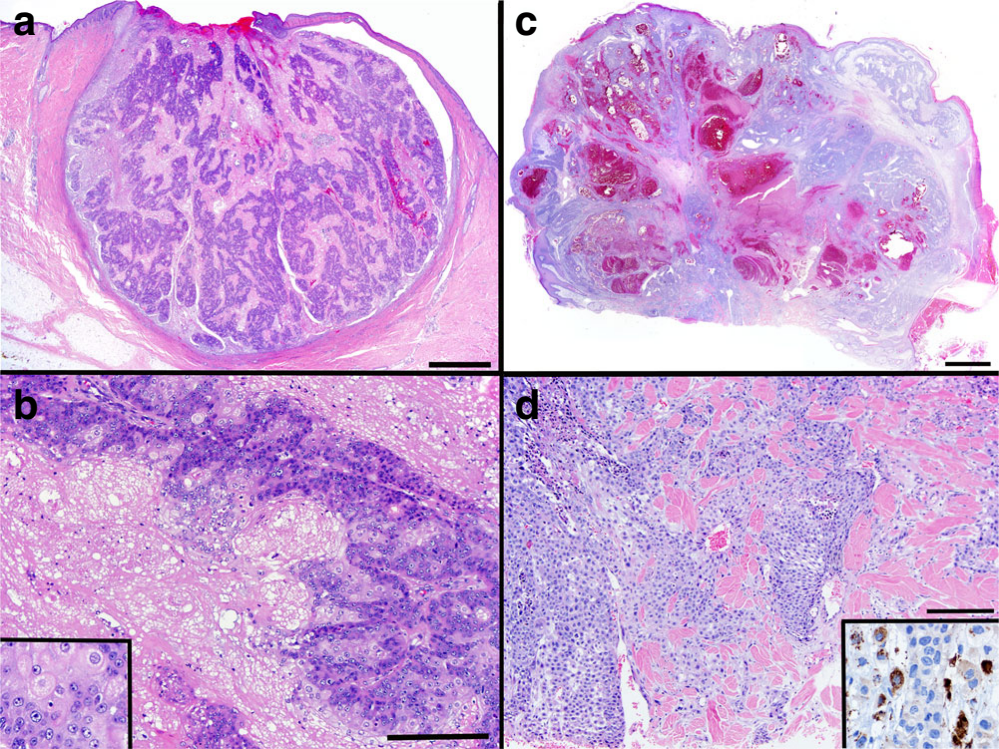
UV-damage class sebaceous carcinomas show poorer differentiation and more infiltration. MSI sebaceous carcinomas have well-circumscribed edges (**a**) with moderate to well-developed sebaceous differentiation in the form of sebaceous gland formation (**b**) and mature, lipid-rich sebocytes (**b**, inset). UV sebaceous carcinomas have more poorly differentiated features (**c**), often requiring adipophilin immunostaining to confirm sebocytic origin (**d**, inset). They frequently have infiltrative features and tumor necrosis (**d**). Scale bar (**a**) and (**c**) 1 mm, (**b**) and (**d**) 0.1 mm

**Fig. 4:**
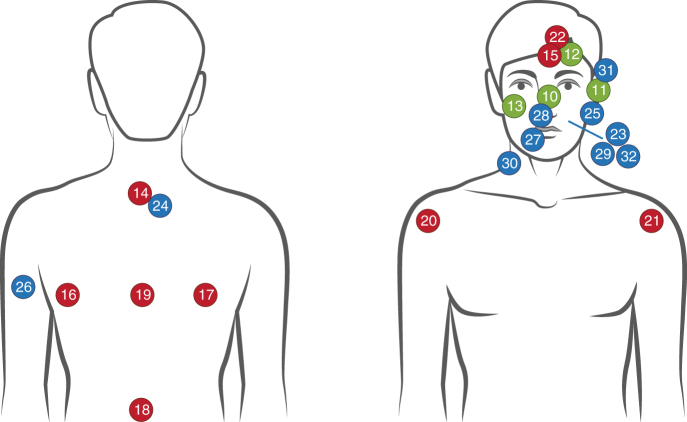
MSI sebaceous carcinomas are more prevalent on truncal skin. Anatomic locations of 23 cutaneous SeC in this study are depicted (the nine ocular SeC are not shown, to improve the presentation). MSI tumors are shown in red, UV-damage tumors in blue, and pauci-mutational cancers in green. Image created using Adobe Illustrator CC

We examined the clinical and histopathologic characteristics of tumors in each of these three classes. The mean age of patients developing UV SeC was 83.1 (7.82), compared to 69.6 (15.2) for MSI and 72.7 (7.26) for pauci-mutational SeC. These differences are significant using a one-way analysis of variance at a *p* value of 0.017. Uniformly, MSI tumors showed greater differentiation (six well-differentiated, three moderately differentiated, Fig. 3a, b) and all nine had well-circumscribed (non-infiltrative) borders. We also found that seven of nine MSI tumors arose on the trunk, a site almost completely spared in the other two classes (Fig. 4). In contrast, UV class tumors were more poorly differentiated (eight moderate, two poor, Supplementary Data 1) and 70% exhibited an infiltrative growth pattern (*χ*^2^ test *p* value = 0.003, Fig. 3c, d).

All UV signature tumors showed histopathologic evidence of moderate to severe sun damage. Prominent squamous differentiation was present in only two tumors, both of the UV signature class. Of the pauci-mutational class, nine were ocular and four were cutaneous on facial skin (Fig. 4). Two (both extra-ocular) were well-differentiated, similar to SeC in the MSI class. Two of the extra-ocular pauci-mutational tumors had histopathologic evidence of moderate to severe sun damage and infiltrative growth patterns. The nine pauci-mutational ocular SeC were all moderately differentiated and showed scant evidence of chronic sun damage, consistent with development on sun-protected epithelium of the inner eyelid.

### SeC driver genes are specified by mutational class

**Fig. 5:**
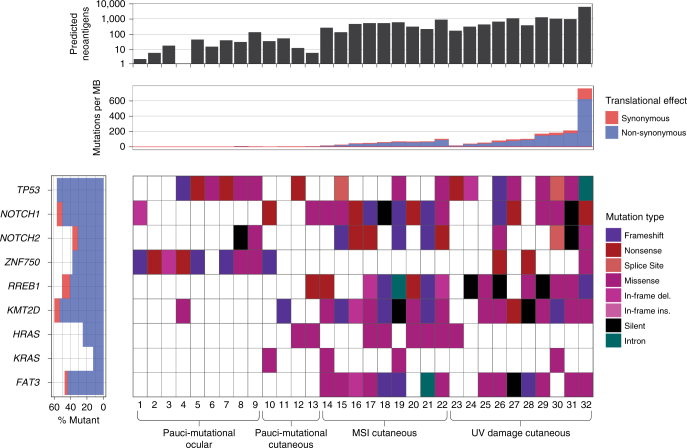
Ocular sebaceous carcinomas acquire truncating *ZNF750* mutations, while UV-damage class tumors show lower prevalence of *HRAS/KRAS* and *RREB1* mutations and acquire higher neoantigen burden. SeC are arranged by mutational subclass on *x* axis and arranged from 1 to 32 from left to right. Selected recurrently mutated genes are shown on a per sample basis, with mutation type coded in color. The single most deleterious mutation type in a sample is displayed, depicted from top (frameshift) to bottom (intron) in the key. The predicted neoantigen burden is displayed by column graph at top of the figure, showing that UV-damage class tumors generally substantially exceed the reported threshold for response in lung cancer^20^

In order to identify the recurrently mutated genes in our SeC, the MuSIC analysis framework was applied^13^. We restricted this analysis to somatic changes occurring at a minimum allele frequency of 20% to reduce the impact of the high background mutation rate and enhance for positively selected variants. Only 11 genes with a convolution test false discovery rate of <1 × 10^*×*−5^ showed both multiple point mutations and indels in our series, including *TP53*, *NOTCH1*, *NOTCH2*, *ZNF750*, *RREB1*, *KMT2D*, and *FAT3* (Fig. 5, Supplementary Data 4). *KM2TD* and *NOTCH1* are known epithelial tumor suppressors affecting epigenetic programming and differentiation, and mutation of *TP53* and *NOTCH1/2* is classically associated with SCC. Although the sebaceous gland epithelium believed to give rise to SeC resembles the germinative differentiation of basal cell carcinomas (BCC), the mutation spectrum of SeC more closely resembles that of cutaneous SCC. No nonsense mutations were observed in *PTCH1*,* SMO*,* SUFU*, or other Hedgehog signaling pathway genes typically mutated in BCC.

We then re-examined individual gene mutations in the context of mutational classes we discovered in SeC (Fig. 5). Truncating *NOTCH1* mutations were notably excluded from ocular SeC (1/9 harbored non-synonymous mutations) while 8/23 of the remaining tumors acquired a nonsense or frameshift mutation (Fig. 5). In contrast, 9/13 pauci-mutational SeC acquired mutations in *ZNF750*, including 6 nonsense and frameshift mutations, compared to just 2/19 truncations in the remaining SeC. Only 2/19 of the UV signature and ocular SeC harbored activating hotspot *KRAS* or *HRAS* mutations, as opposed to 10/13 of the MSI and pauci-mutational cutaneous classes (*χ*^2^ test, *p* value = 0.0002). Similarly, 4/9 MSI tumors harbored truncating mutations in *RREB1*^14^, compared to only 2/23 of the non-MSI cancers.

### UV SeC transcriptomes resemble those of SCC and BCC

**Fig. 6:**
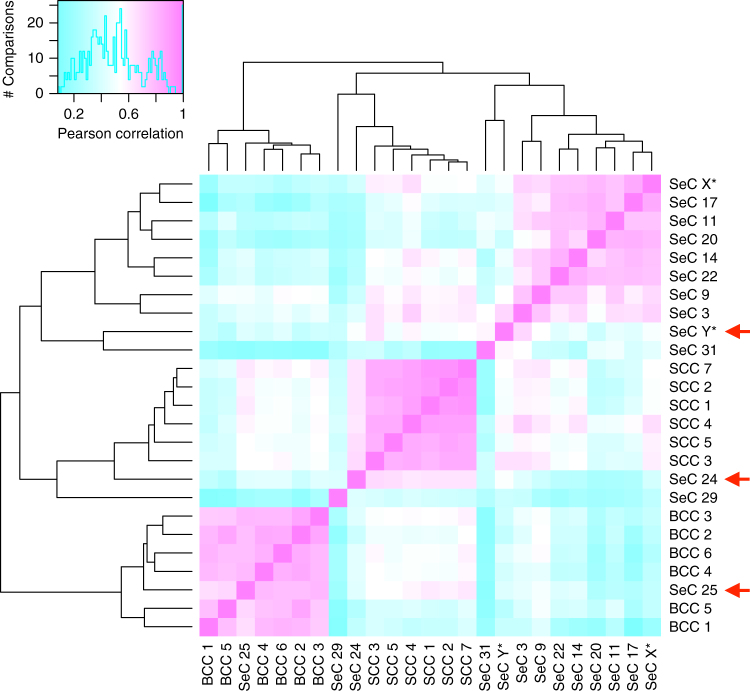
UV-damage class and pauci-mutational but not MSI sebaceous carcinomas share transcriptomic similarities with SCC and BCC. Pearson correlation comparisons for 500 genes of highest variance for 5 UV class (samples 24, 25, 29, 31, Y*), 5 MSI (14, 17, 20, 22, X*), and 3 pauci-mutational SeC (3, 9, 11) along with 6 each of spontaneously arising SCC and BCC. Correlations are coded from low (blue) to high (pink) as shown on histogram of sample–sample comparisons in upper left. Sample X* is a verified MSI germline SeC and Y* is a heavily UV-damaged SeC not included in exome analysis because of lack of normal control tissue. Red arrows denote UV SeC samples with high degrees of transcriptional similarity to specific SCCs and BCCs

All classes of SeC acquire recurrent mutations identified in cutaneous SCC, including *TP53, KMT2D*^15, 16^, and *NOTCH1*/*NOTCH2*^17^. However the squamous differentiation and infrequency of *KRAS*/*HRAS* activation in the UV subclass led us to hypothesize that these tumors may display epigenetic similarity with SCC, which share transcriptional patterns across their many subtypes^18^. RNA sequencing was performed on five UV class, four MSI, and four pauci-mutational SeC, along with six each of spontaneously arising cutaneous SCCs and BCCs (Fig. 6). Strikingly, heavily UV-damaged samples SeC 24, 25, and Y shared transcriptomic patterns resembling those found in SCC and BCC, and none of the five clustered with MSI or pauci-mutational SeC. SeC 24 RNA expression was more strongly correlated with all six SCCs than any SeC (*t* test *p* value 1*e* − 6). Similarly, SeC 25 RNA expression correlated with all six BCCs more closely than with any SeC (*t* test *p* value = 1*e* − 6). SeC Y had higher average correlation with SCC samples than SeC; its transcripts also differentiated it significantly from BCC (*t* test *p* value = 0.009). Pauci-mutational SeC 3 and 9 also showed some similarity to SCC, but clustered most strongly with MSI SeC. None of the eight pauci-mutational and MSI SeC profiled showed sufficient transcriptional similarities to classify them with keratinocytic malignancies.

### UV and MSI SeC harbor high neoantigen burdens

The different mutation rates between SeC subclasses seemed likely to proportionately impact predicted neoantigen load, potentially affecting immunotherapy response. As expected, UV-damage tumors harbored the largest numbers of predicted neoantigens, followed by MSI tumors, then the pauci-mutational SeC (Fig. 5, Supplementary Data 5). The total tumor mutation burden per sample exceeded 100 for all 32 tumors, which has been reported as a threshold for response to CTLA-4 blockade in melanoma^19^. The total predicted neoantigen burden exceeded 100 for 24 of 32 samples, the threshold for clinical benefit reported in non-small cell lung cancer^20, 21^. Clonality of predicted neoantigens ranged widely in our SeC series, from 0 to 100% and did not show differences by mutational class. Eleven out of 32 samples harbored predicted neoantigens that were at least 50% clonal.

## Discussion

Here, we report three distinct mechanisms by which SeC accumulate somatic mutations. Indel-rich MSI SeC appear to be initiated by inactivating mutations in mismatch-repair genes (which can be either germline or somatic), while SeC with UV-damage-associated mutational signatures develop on heavily sun-damaged skin. Although sebaceous neoplasms have been reported anecdotally to harbor somatic mutations in mismatch-repair genes^8, 22^, our results show that virtually all MSI class SeC arise from either a germline or somatic mutation in this pathway. A third, previously undescribed pauci-mutational class is primarily represented by facial SeC. In fact, all nine of the ocular SeC in our series displayed this pattern.

Remarkable mutual exclusivity characterizes the mutation signatures in SeC. Even in MSI SeC that arise on sun-exposed skin, mutations characteristically associated with UV damage are virtually absent. It is conceivable that all SeC originate from sebocytes, with the UV class undergoing dedifferentiation and acquiring keratinocytic features. However, the considerable UV-damage burden in this subset (Fig. 1) militates against this hypothesis. UV class SeC are not found more superficially in the dermis than MSI tumors, where they could acquire UV mutations during a later growth phase. In fact, MSI SeC, which show the most distinctive features of sebaceous differentiation, acquire much lower levels of UV-associated mutagenesis, as one would expect of sebocytes located in the mid-dermis. A hypothesis of divergent evolution of SeC subtypes also fails to explain why, like MSI SeC, UV subclass tumors acquire truncating *NOTCH1* mutations, aberrations that should restrict keratinocytic differentiation^23^.

We therefore favor an alternative model in which poorly differentiated UV-damage SeC, like cutaneous SCC, arise from a subpopulation of more superficial keratinocytes vulnerable to UV mutagenesis, either from the epidermis or superficial portion of the folliculosebaceous unit. In this model, UV class SeC may acquire sebocytic differentiation because of specific somatic alterations or the epigenetic state of the cell of origin. This hypothesis is consistent with the scarcity of UV-damage SeC on the trunk (Fig. 4) and helps explain why tumors with prominent squamous differentiation are found exclusively in the UV class. A keratinocytic origin of UV SeC also accounts for relatedness of transcriptomes of UV SeC and SCC and BCC, which diverge substantially from MSI and pauci-mutational SeC.

In contrast, MSI SeC invariably arise through inactivation of mismatch-repair genes. The predilection of these cancers for sebocyte-rich skin (Fig. 4) raises the question: do all cells of epithelial lineage acquire stochastic mutations in mismatch-repair genes, with sebocytes particularly susceptible to subsequent hypermutation and tumor evolution? Or do sebocytes permit higher mutation rates that stochastically inactivate repair genes? The answer is germane to the anatomic distribution of other skin cancers. For example, in multiple hereditary infundibulocystic BCC syndrome, a germline mutation inactivates one copy of the *SUFU* repressor of the Hedgehog signaling pathway^24^. Basal cell cancers arise in affected individuals when the wild-type *trans* copy is inactivated by small deletions. However, these cancers arise predominantly in the central face and genital area. Either cells at these sites are somehow more prone to deletion events, or they are more vulnerable to tumorigenesis once such events occur. We also report the *RREB1* gene is frequently mutated in MSI SeC. A transcription factor that functions in adipocytic differentiation^14^*, RREB1* has not been previously implicated as a tumor suppressor gene in human cancer. We detected robust RREB1 protein expression in adult sebaceous glands; its role in differentiation and the consequences of its deactivation in SeC remain to be investigated.

Pauci-mutational SeC occur exclusively on the face (13/13), and show no dominant mutational signature. They may represent relatively sun-shielded epithelial cells that by chance acquire driver mutations because of the modest mutagenesis of normal aging. Interestingly, the *ZNF750* transcription factor acquired truncating mutations in 6/13 of these tumors. ZNF750 is required for normal epithelial homeostasis^25^ and has recently been explored as a lineage-specific tumor suppressor in SCC^26^. Its striking rate of inactivation (frameshift or nonsense mutations) in ocular SeC (5/9 samples) has not been reported. The recent Tetzlaff et al.^8^ targeted sequencing study in ocular SeC did not appear to assess for *ZNF750* mutations in their panel of genes. Our data suggest that ZNF750 inactivation is critical for tumorigenesis in ocular, and perhaps facial SeC more generally. Only two of the 19 UV and MSI SeC were mutated for *ZNF750*. Conversely, 0/9 ocular SeC acquired truncating *NOTCH1* mutations, compared to 5/9 of the MSI class. The class specificity of *NOTCH1*,* RREB1*, and *ZNF750* mutation supports the theory that epigenetic differences in epithelial sites underlie distinct mechanisms of SeC tumorigenesis. We did not detect activating *PIK3C* or inactivating *PTEN* mutations in either ocular or extra-ocular SeC, but such mutations may contribute a selective advantage in a minority of cases^8^.

Our data illustrates how cancers grouped by histopathologic similarity actually arise from different mutational mechanisms, likely acting on distinct cells of origin. These differences reframe clinical approach. Investigations for germline mismatch-repair defects will rarely prove successful in the context of UV class SeC. MSI SeC may also behave indolently, much like their visceral counterparts^27^, mitigating the urgency of follow-up and surveillance. This classification also impacts therapeutic approaches to those cancers that metastasize. Given the reported correlation between poor differentiation and metastasis^4^, it is likely that disseminated disease either falls into a highly mutated (UV-damage) class amenable to immunotherapeutics, or a pauci-mutational class, including most ocular SeC, predicted to respond less robustly. In our series, poorly differentiated UV SeC showed predicted neoantigen burdens at times exceeding 500 per sample, >50% of them clonal in some cases, well above published thresholds for reliable treatment response.

## Methods

### Sample inclusion and histopathologic classification

With UCSF IRB approval and a waiver of informed consent, we screened the UCSF dermatopathology tissue archives by diagnostic code for SeC from 2007 to 2015 that had available tumor and normal skin remaining in the paraffin block(s). The diagnosis of SeC was confirmed by a board-certified dermatopathologist (JPN). For any tumors in which sebocytic differentiation was questionable, adipophilin immunoperoxidase staining was used to confirm sebaceous differentiation. Any tumors in which the diagnosis was uncertain after adipophilin stainings were excluded. Each tumor was classified by the degree of differentiation (e.g., poorly, moderately, well differentiated) as manifested by sebaceous gland organization and the number of mature sebocytes present. High-grade cytologic atypia and squamous differentiation were noted if present. Solar elastosis was graded as minimal or moderate to severe, and tumors were assessed for infiltrative growth pattern at the edges of the neoplasm.

### Immunostaining

Formalin-fixed paraffin-embedded (FFPE) sections of 4-µm thickness were stained with the following immunoperoxidase stains: adipophilin (393A-18, Cell Marque, Rocklin, CA, predilute on Leica Bond); MLH1 (PA0610, Leica); MSH2 (IR08, Dako).

### DNA extraction and exome sequencing

Ten 5-µm tissue sections were cut using standard microtomy techniques. The top section was subjected to hematoxylin and eosin staining and reviewed by a board-certified pathologist who identified and marked regions of malignancy. Using the marked-up image as a guide, tissue sections were macrodissected to collect the desired regions. Each collected cell population was then extracted using the Qiagen QIAamp DNA FFPE Tissue Kit for DNA and RNeasy FFPE Kit for RNA. Extracted material was then quantified using a Qubit fluorometer.

DNA-seq libraries were captured to exome regions using xGen Exome Research Panel v1.0 (IDT), and libraries were prepared using the KAPA Hyper prep kit. DNA libraries were sequenced to a target depth of ×200 for tumor sample, ×100 for normal samples on the Illumina HiSeq platform.

RNA-seq libraries were prepared using the KAPA Stranded RNA-Seq Kit with RiboErase (Kapa Biosystems, Wilmington, MA) and sequenced to a target depth of 200-M reads on the Illumina HiSeq platform (Illumina, San Diego, CA). RNA samples were aligned to RefSeq build 73 transcriptome using Bowtie2 v2.2.6 and quantified using RSEM v1.2.25^28^.

### Exome variant calling

Raw sequencing data in Illumina’s fastq data format was converted into fastq files with base quality scores encoded in the Sanger basecall format. Next, the reads were aligned using the Burrows–Wheeler Aligner (BWA)^29^. This aligner is based on the Burrows–Wheeler transformation, aligns paired-end reads and handles indels robustly. The output of BWA are the aligned reads in sequence alignment/map (SAM) format. Reads stored in SAM format were then converted to the binary SAM (BAM) format using the samtools software^30^. Once reads were in the sorted and indexed BAM file format, position based retrieval of reads was expedited and data storage requirements were minimized. Next, to remove erroneous mutation calls due to PCR duplication, all duplicate reads were removed using samblaster^31^. After removal of the duplicate reads, the base quality scores were recalibrated using the CountCovariates and TableRecalibration tools included in the Genome Analysis Toolkit software, also developed by the Broad Institute (https://www.broadinstitute.org/gatk/).

The tumor and matched-normal BAM files were analyzed for SNVs with the following method. Sequenced bases with ≥8 unique (nonduplicate reads) with mapping quality ≥20 in both tumor and matched-normal were used to compute the likelihoods of all possible genotypes (AA, AT, AC, and so on) using a mapping quality-based error model^29^, available in the samtools source code. If fewer than two reads support any non-reference allele at the current position, then the position was deemed homozygous reference and no further analysis was performed. The genotype likelihoods were used in a Bayesian model used by the SomaticSniper method^32–34^, incorporating a prior probability on the reference, the fraction of heterozygous positions in the human genome, the probability to convert the normal genotype to the tumor genotype. Each tumor/normal genotype pair was scored using this model. The genotype pair with the highest likelihood, given the data, was chosen as the most likely tumor and normal genotype. Any position that was determined to be homozygous for the reference allele in both tumor and normal was not further analyzed.

If tumor and normal genotypes were identical, then the variant was classified as germline. Instances where the normal genotype was heterozygous and the tumor genotype was homozygous suggest not new point mutations but regions of loss-of-heterozygosity (LOH), and such variants were so classified. For variants classified as either germline or LOH, the log-likelihood of the paired genotype was used to compute a Phred-scaled quality/confidence of the germline variant. All other variants were classified as a somatic mutation and their somatic score (SS) was calculated^32^. For any position where the tumor and/or normal genotype was not homozygous for the reference allele, a number of metrics were computed, including number of total reads, number of allelic reads, average base and mapping quality, number of reads with mapping quality = 0, number and quality sum of mismatches in reads with variant or reference allele, the distance of the variant to the 3′ end of the read, and number of reads aligned to the forward/reverse strand. All putative variants and their associated metrics were converted to the variant call format (VCF) and the following filters applied^32^: conf: genotype quality or SS ≥15 dp: total depth (DP of normal + primary) ≥4; mq0: number of mapping quality = 0 reads <5; sb: mutant allele strand bias *p* value >0.005 (binomial test); mmqs: quality sum of mismatches (per read) ≤20; amm: average number of mismatches (per read) ≤1.5; detp: fractional distance to 3′ <0.2 or >0.8; ad: mutant allele depth in tumor ≥4; gad: mutant allele depth in normal ≤3; ma: read support is ≥2 for two or more alternate alleles at this position^32^. A single VCF file was produced for each tumor sample.

### Calling copy-number variants

For copy-number variants (CNVs), referred to here as CNVs, average tumor vs. matched-normal relative coverage and standard deviation were calculated for each captured exon by dividing read depth measured in the tumor by the read depth in the matched-normal for each position within the exon. Exons that were insufficiently covered, average read depth <5 reads, in both tumor and matched-normal were removed from the remaining analysis.

Average majority allele fraction in both tumor and matched-normal was computed for each segment with at least one heterozygous SNP in the normal tissue:

$${\mathrm{AF}}_m^t = \frac{{{\mathrm{DP}}_{{\mathrm{major}}}^t}}{{{\mathrm{DP}}_{{\mathrm{total}}}^t}},\,{\mathrm{AF}}_m^n = \frac{{{\mathrm{DP}}_{{\mathrm{major}}}^n}}{{{\mathrm{DP}}_{{\mathrm{total}}}^n}},$$

where ${\mathrm{DP}}_{{\mathrm{major}}}^t$ and ${\mathrm{DP}}_{{\mathrm{major}}}^n$ are the read depths of the germline allele with the greatest read support and ${\mathrm{DP}}_{{\mathrm{total}}}^t$ and ${\mathrm{DP}}_{{\mathrm{total}}}^n$ are the total read depths at the position in the tumor and matched-normal, respectively. Relative coverage is determined simply by dividing the coverage observed in tumor by the matched-normal’s coverage, i.e., ${\mathrm{RC}} = {\mathrm{DP}}_{{\mathrm{total}}}^t/{\mathrm{DP}}_{{\mathrm{total}}}^n$. The standard deviation of AF_*m*_ estimates was computed for segments featuring at least three heterozygous SNPs. Only dbSNP sites with sufficient read support in the tumor and matched-normal $({\mathrm{DP}}_i^t \ge 10,{\mathrm{DP}}_i^n \ge 20)$ deemed heterozygous ($0.25 \le {\mathrm{AF}}_i^n \le 0.75$) are considered when computing majority allele fraction estimates.

To determine CNVs, the exon-level statistics computed above were iteratively aggregated into larger segments in an agglomerative process similar in spirit to hierarchical clustering. In the first round, every pair of neighboring exons was analyzed. Neighboring exons that did not have significantly different relative coverage and ${\mathrm{AF}}_m^t$ (only for exons with heterogeneous SNPs) estimates (*p* value >0.95, two-sample Student’s *t* test) were merged into a single segment. The average relative coverage for the new segment was calculated as the base-pair count adjusted averages and standard deviations of the two individual exons measurements, while ${\mathrm{AF}}_m^n$ and its standard deviation were recomputed for the new segment using the same procedure described above. This procedure was continued with neighboring segments using the above method for three rounds.

The relative coverage estimates of all segments were centered to the median of the entire genome, which was assigned the value of 1.0, signifying the normal copy-number state. The skew in ${\mathrm{AF}}_m^t$ estimates caused when the majority allele’s read support is due to sampling bias instead of an underlying imbalance in allele copy number was corrected by subtracting out the ${\mathrm{AF}}_m^n$ estimated in the matched-normal sample. However, since this bias is only present in regions where both alleles have equal copy number, the correction is made using the following equation:

$${\mathrm{AF}}_{m,{\mathrm{corr}}}^t = {\mathrm{AF}}_m^t - \left( {{\mathrm{AF}}_m^n - 0.5} \right)e^{ - 0.5\left( {\frac{{{\mathrm{AF}}_m^t - {\mathrm{AF}}_m^n}}{{0.05}}} \right)^2}.$$

The above copy-number segments are then used to estimate the amount of normal contamination, *α*, and tumor ploidy that can be used to transform the relative coverage estimates into allele-specific, integral copy-number states where possible.

Best-fit parameters for *α* and tumor ploidy are found by gradient descent. Each round of gradient descent is initialized with random values for *α* and ploidy and attempts to maximize the joint log-likelihood of the relative coverage, rc, and majority allele fraction, af, estimates weighted according to segment size across the 22 autosomes. Gradient descent is performed in this manner for a minimum of 10 times, and the best-fit parameters across all rounds are reported.

In the joint log-likelihood calculation, a set of common allelic states are used to determine the expected relative coverage and majority allele fraction values for each state, given *α* and tumor ploidy. These states include commonly altered states such as single-copy gain (2, 1), LOH (1,0), and copy-neutral LOH or CN-LOH (2, 0), where the numbers in parentheses are the majority and minority allelic copy numbers, (*A*, *B*), that describe an allelic state. Additionally, less common states such as balanced amplification (2, 2) and subclonal states representing a 50/50 mixture of subclones with and without an altered allelic state are also used.

Tumor ploidy is recalculated using the best-fit parameters to transform the original relative coverage estimates into tumor copy number. Ploidy is then calculated as the average of tumor copy number across the whole genome, weighted by the normalized genomic length of each segment. Any copy-number segment that was normal (1, 1) or that deviated significantly (two-sample *t* test *p* value <0.05) from an integral state was not included in the downstream analysis, and any segment larger than 2.5 megabases is detailed in the supplemental tables.

### Microsatellite instability sequence analysis

Instability of microsatellite repeats is estimated using the method described here^10^. A set of 2848 microsatellites consisting of homopolymer repeats were analyzed for an increase in the number of length polymorphisms in both tumor and matched-normal (if available) sequenced. The background mean (*μ*) and standard deviation (*σ*) of the number of length polymorphisms for each microsatellite locus were computed across ~5000 blood and solid normal exomes sequenced by TCGA comprising 18 different cancer types. Loci covered by fewer than 30 reads are excluded from the analysis. For each microsatellite locus, the number of differently sized repeats are counted for each sample. Repeats with read support exceeding 5% of the read support of the maximally supported repeat are tallied for a total count of differently sized repeats, *n*. The total number of unstable microsatellites is counted in each sample, where a given microsatellite *i* is deemed unstable if *ni* > *μi* + 3*σi*. The percentage of unstable loci is calculated for the tumor. If the matched-normal sample is available, its estimated percentage of unstable loci is subtracted from the tumor’s estimate in order to remove sample-specific variation in microsatellite stability. If the tumor sample’s (relative) percentage of unstable loci is 15% or higher, then it is judged as having a high degree of MSI.

Disruptive alterations to DNA repair genes (*MLH1*, *MSH2*, *MSH6*, *PMS2*) are presented in this analysis. Alterations are restricted to: somatic and germline nonsense SNVs, somatic, and germline frameshifting insertions or deletions, and somatic gene losses.

### Mutation signature analysis

The bases directly adjacent to the mutated site are used to determine the genomic context of the site, which can help to determine if a particular mutagen (e.g., tobacco smoke, exposure to ultraviolet light) or mutational process is active in the sample. Classification of point mutations in our study among the 30 signatures defined by the Signatures of Mutational Processes in Human Cancer database (http://cancer.sanger.ac.uk/cosmic/signatures) was calculated using non-negative matrix factorization on the counts of mutated triplets identified in the tumor sample^9^. “Active” signatures are those that contributing at least 100 mutations or >25% of the total mutations in the sample.

### Statistical analysis of SeC subclass and histopathology

Histopathologic parameters were assessed as dichotomous variables with *χ*^2^ analysis. In the case of histopathologic differentiation, low and moderate categories were combined and compared against well-differentiated tumors. A *p* value <0.05 was considered statistically significant.

### Neoepitope prediction analysis

Potential neoepitopes were generated by identifying all mutations that caused a protein change within exonic regions. A sliding window of nine amino acids around the mutation site was used to identify all possible sequences arising from a mutation. RNA-seq was used where available to identify only expressed neoepitopes. NetMHC 3.4 was used to identify expressed neoepitopes that were capable of being bound by the matched patient human leukocyte antigen (HLA typing). HLA typing was done by aligning sequences from HLA regions against sequences in the IMGT/HLA database^35^.

### RNA extraction and expression analysis

RNA-Seq libraries were prepared for the tumor sample using KAPA Stranded RNA-Seq with RiboErase kit and sequenced on the Illumina sequencing platform using two library preparations, with each library targeted for 100-M sequencing reads. RNA sequencing reads were aligned by bowtie2 using default parameters to the RefSeq transcriptome and analyzed by RSEM. Gene-level transcripts per million (TPM) estimates were transformed via log2 (1 + TPM) before further correlation analysis. The Pearson correlations reported here were calculated on the 500 genes with highest variance. The correlation trends were verified on two further gene sets: all genes with variance >1.0, and the full transcriptome. Hierarchical clustering was performed on the correlation matrix in R, using the default parameters of the heatmap.2 package from gplots version 3.0.1.

### Data availability

Sequence data has been deposited at the European Genome-phenome Archive (EGA), which is hosted by the EBI and the CRG, under accession number EGAS00001002869.

Further information about EGA can be found on https://ega-archive.org and “The European Genome-phenome Archive of human data consented for biomedical research”^36^. All other data are available from the authors upon reasonable request.

## Electronic supplementary material

Description of Additional Supplementary Files

Supplementary Dataset 1

Supplementary Dataset 2

Supplementary Dataset 3

Supplementary Dataset 4

Supplementary Dataset 5
