# Supplementary material for: Cell of origin and mutation pattern define three clinically distinct classes of sebaceous carcinoma
Source: Nat Commun. 2018 May 14;9:1894. doi: 10.1038/s41467-018-04008-y (PMC5951856; doi:10.1038/s41467-018-04008-y)
Supplement: Supplementary file 1 — Description of Additional Supplementary Files [file 41467_2018_4008_MOESM1_ESM.pdf]

Supplementary Data Legends

Supplementary Data 1. **SeC sample overview.** Listed are sample number, age of patient at excision, anatomic site of cancer, histopathology, other stains, solar elastosis, circumscription, total mutations determined from exome sequencing, mutations/megabase, mutational origin class of cancer, total SSNVs, total indels; mutations in mismatch repair genes, *ZNF750*, *HRAS*, and *KRAS*.

Supplementary Data 2. **SeC mutation catalog.** Each worksheet represents an independent sample. Mutations are listed by gene, type, class, protein change, chromosome and start/stop position, quality score, alternate allele, reference allele, read depth of alternate allele, read depth of all alleles, alternate allele percentage, number of COSMIC references for base change, listing in the Cancer Gene database, and matches in dbSNP.

Supplementary Data 3. **SeC copy number variant catalog.** Each worksheet shows copy number events > 2.5 megabases in size, as determined by calculation of tumor copy number across the whole genome, weighted by the normalized genomic length of each segment. Samples 7, 12, and 13 do not harbor CNVs.

Supplementary Data 4. **SeC MuSIC analysis significant mutated genes.** The MuSIC analysis framework3 was applied to somatic changes occurring at a minimum allele frequency of 20%. The 24 genes with lower than a false discovery rate of $< 1 \times 10^{-3}$ are listed here.

Supplementary Data 5. SeC neoantigen catalog. Neoantigen burden, percent clonality of neoantigens, and total mutation burden shown for each SeC, calculated as detailed in Methods.
